# Spatial Analysis of Placentae During Congenital Cytomegalovirus Infection Reveals Distinct Cellular Profiles in Immune Cells and Trophoblasts

**DOI:** 10.1101/2025.04.04.647170

**Published:** 2025-04-10

**Authors:** Elise Sintim-Aboagye, Huy Quang Quach, Will Sherman, Sheila Farnan, Kamila Otrubova, Namisha Verma, Dawn Littlefield, Sohan Punia, Erica Johnson, Mark Blackstad, Mark R. Schleiss, Andrew P. Norgan, Clive M. Gray, Elizabeth Ann L. Enninga, Rana Chakraborty

**Affiliations:** 1 Department of Obstetrics and Gynecology, Mayo Clinic, Rochester, MN, USA;; 2 Mayo Clinic Vaccine Research Group, Department of Internal Medicine, Mayo Clinic, Rochester, MN, USA;; 3 Department of Quantitative Health Sciences, Mayo Clinic, Rochester, MN, USA;; 4 Department of Pediatric and Adolescent Medicine, Mayo Clinic, Rochester, MN, USA;; 5 Department of Microbiology, Biochemistry, and Immunology, Morehouse School of Medicine, Atlanta, GA, USA;; 6 Department of Pediatrics, Division of Pediatric Infectious Diseases, University of Minnesota Medical School, Minneapolis, MN USA;; 7 Department of Laboratory Medicine and Pathology, Mayo Clinic, Rochester, MN, USA; 8 Division of Immunology, Department of Biomedical Sciences, Biomedical Research Institute, Stellenbosch University, Cape Town, South Africa

**Keywords:** congenital, cytomegalovirus, placenta, immune cells, trophoblasts, proteomics, transcriptomics

## Abstract

Cytomegalovirus (CMV) is the most common cause of birth defects by an infectious agent. Approximately 10% of infants with congenital CMV (cCMV) infection are symptomatic. Infected infants can exhibit long-term effects such as sensorineural hearing and vision loss and neurodevelopmental delay. To date, the mechanisms by which cCMV infection results in symptomatic disease are incompletely understood. The placenta has been implicated as a main thoroughfare for vertical transmission, as both placental immune cells and trophoblasts can be infected by CMV. The goal of this study was to spatially investigate changes in genes and proteins from immune cells and trophoblasts during cCMV infection. Utilizing the NanoString GeoMx Digital Spatial Profiler, we noted that both immune cells and trophoblasts in CMV^+^ placentae exhibited increased expression and upregulation of immune activation receptors and pathways. Pro-apoptotic proteins were decreased in CMV^+^ placentae, as were transcripts associated with cell death pathways. Spatially, immune cells infiltrating into CMV^+^ placental villi had more CD4^+^ T cells expressing cell death markers than those T cells in the decidua (p = 0.002). In contrast, the decidua exhibited a CD8+ T cell abundance with far less upregulation of immune activation receptors than in the villi (p=0.03). These data can inform and direct future research into the immune mechanisms CMV uses to infect, evade, and vertically transmit the virus to the fetus.

## 1. INTRODUCTION

Congenital infection with CMV is the most common infectious cause of birth defects globally, impacting an estimated one million infants annually.^1,2^ According to the Centers for Disease Control and Prevention (CDC), cCMV infection is estimated to occur in 1 out of 200 live births and nearly 1 in 3 children are infected, usually by horizontal transmission, with CMV by age 5 in the United States.^3^ Approximately 10% of cCMV infections in newborns are symptomatic, some exhibiting multi-system disease associated with considerable morbidity and mortality.^4^ Even in the absence of symptomatic illness at birth, infants with cCMV often fail to achieve comparable health outcomes compared to uninfected infants, exhibiting an increased incidence of sensorineural hearing loss, impaired psychomotor development, and altered growth.^5–8^ Previous studies show that women of childbearing age with preconception immunity to CMV are 69% less likely to give birth to an infant with cCMV infection than those who are CMV seronegative at the onset of pregnancy.^9^ These observations suggest that prevention strategies focused on a vaccine capable of inducing CMV immunity in women of childbearing age are likely to be beneficial.^10^ However, there exists challenges for the development of effective CMV vaccines and none are currently licensed, reflecting our incomplete understanding both of CMV pathogenesis^11^ and of the correlates of protective immunity for the maternal-placental-fetal unit.^12^

Transmission of CMV can occur horizontally through infected bodily fluids, and vertically during intrauterine life through the placenta, via blood and vaginal secretions at the time of delivery, and postnatally through breastmilk.^13,14^ Of these routes, vertical transmission via the placenta represents the most significant route for cCMV infection.^15^ Still, there exists significant gaps in our understanding of CMV pathophysiology and its interaction with cells in the placenta. Here, we aimed to characterize alterations in placental cells from infected pregnant people using novel spatial-omics, shedding light on CMV pathogenesis at the maternal fetal interface. In this study, we examined the proteomic and transcriptomic characteristics of placental tissue using the NanoString Digital Spatial Profiler (DSP). By obtaining placental tissue from pregnant people who gave birth to infants with cCMV and matched controls, we addressed the following key questions: 1) How does gene and protein expression differ in CMV infection of the placenta by cell type? 2) Are there spatial differences in gene and protein expression based on the placental compartment (villi versus decidua)?

## 2. METHODS

### 2.1. Ethics Statement

The Institutional Review Board (IRB) of Mayo Clinic approved this study (IRB #20–012379). All study participants provided informed consent allowing their placental tissue and medical records to be reviewed for this study.

### 2.2. Placental Tissue Samples

Residual formalin fixed paraffin embedded (FFPE) placental tissue from 7 participants who underwent Cesarean section were obtained from the Mayo Clinic Tissue Repository and sectioned by the Mayo Clinic Pathology Core. Three of these samples were from pregnancies with a confirmed case of a primary CMV infection during pregnancy with infectious villitis pathology, and 4 were uncomplicated pregnancies undergoing elective Cesarean section with normal placental anatomy and no documented underlying infections. Polymerase Chain Reaction (PCR) was performed to confirm the CMV cases were positive for CMV viral DNA (Supplemental Table 1).

### 2.3. Transcriptomic Profiling by NanoString GeoMx

For GeoMx DSP slide preparation for spatial transcriptomics, we followed the user manual. FFPE placental slides were baked at 60°C for 3 h to remove excess paraffin wax. The slides were then deparaffinized and rehydrated using serial washes of CitriSolv, 100% ethanol, 95% ethanol, and 1× PBS. Target retrieval was performed on the slides by heating up a staining jar containing 1× Tris EDTA to 99 °C in a steamer, adding the slides to the staining jar, and letting them incubate for 5 min. Ribonucleic acid (RNA) targets were exposed by treating the tissue with a 1 μg/mL concentration of Proteinase K followed by fixation with 10% formalin. After pretreatment, tissue slides were incubated overnight with an RNA probe mix (GeoMx Human Whole Transcriptome Atlas; NanoString, Seattle, WA) The next day, stringent washes were performed using a solution of 100% formamide and 4× SSC followed by blocking for non-specific signal incubation. Placental tissue was then stained with the following morphology markers: anti-CD45-AF594 (2B11+PD7/26, 5 μg/mL), anti-panCK-AF532 (AE1+AE3, 2 μg/mL) and SYTO13-FITC (NanoString, 1 μg/mL). Antibodies were purchased from Novus. Slides were loaded onto the NanoString DSP instrument and scanned to visualize whole tissue images. For each tissue sample, a total of 4 regions of interest (ROIs) were selected and collected from each of three tissue types within the placenta: distal villi, stem villi, and decidua. Each ROI was subdivided into areas of illumination (AOIs) based on fluorescent cell-specific markers for CD45 and pan-CK, and serial UV illumination of each compartment used to sequentially collect the mRNA probe barcodes from each cell type.

Each GeoMx DSP sample plus non-template controls (NTCs) were uniquely indexed using the i5 × i7 dual-indexing system (Illumina, San Diego, CA). Four μL of a GeoMx DSP sample was used in a polymerase chain reaction (PCR) with 1 μM of i5 primer, 1 μM i7 primer and 1× NSTG PCR Master Mix. Thermocycler conditions were 37 °C for 30 min, 50 °C for 10 min, 95 °C for 3 min, 18 cycles of 95 °C for 15 s, 65 °C for 60 s, 68 °C for 30 s, and final extension of 68 °C for 5 minutes. PCR reactions were purified with two rounds of AMPure XP beads (Beckman Coulter, Brea, CA) at 1.2× bead-to-sample ratio. The libraries were then sequenced using the Illumina NextSeq2000 P2 (2×50) for a maximum of 800M aligned reads.

### 2.4. Proteomic Profiling by NanoString GeoMx

The method recommended for GeoMx DSP (NanoString) was used in this study. Briefly, 5 μm sections of FFPE placental tissue were adhered to positively charged glass microscope slides and then baked for 3 h at 60 °C in a HERATHERM incubator (Thermo Fisher, Pittsburgh, PA) before tissue deparaffination and rehydration. Antigen retrieval was then performed by boiling the slides in glass staining jars containing citrate buffer for 15 min. Slides underwent a blocking step with Buffer W (NanoString) for 1 h at room temperature and were then incubated overnight with 3 fluorescent antibodies and a panel of 49 oligonucleotide (oligo)-conjugated antibodies (Supplemental Table 2) at 4 °C. Fluorescent antibodies included anti-CD45-AF594 (2B11+PD7/26, 5 μg/mL, Novus)), anti-PanCK-AF532 (AE1+AE3, 2 μg/mL, Novus), and anti-SYTO-FITC (1:200, NanoString).

The slides were scanned on the GeoMx DSP, and 12 ROIs were selected per slide and each ROI was then segmented into AOIs based on the fluorescent antibodies. We selected 4 ROIs from the distal villi, 4 ROIs from the stem villi, and 4 ROIs from the decidua to get a spatial perspective of the impact of CMV throughout differing regions of the placenta. After ROI selection, UV light cleaved the photocleavable oligos off the antibodies, and each AOI was collected in its own well on a 96-well plate, yielding a total of 144 AOIs. After collection, the oligos were hybridized and quantified on an nCounter (NanoString).

### 2.5. Statistical Analysis

Analysis of the data was conducted using the DSP Analysis Suite. The raw data was processed through quality control and normalization strategies that were recommended by the manufacturer. Quality control was based on Field of View Registration, Minimum Surface Area of ROIs, Minimum Nuclei Count within ROIs, Binding Density. Data was normalized using housekeeping genes, including GAPDH, S6, and Histone H3. nCounter reading were compared using linear mixed model analysis with Bonferroni correction within the DSP Analysis Suite. Statistical analyses of GeoMx spatial transcriptomics sequencing data were performed using R version (v4.2.2). Raw Illumina counts were filtered using standard QC threshold settings as recommended by the manufacturer. Counts were normalized to the upper quartile. Processing count data was done using GeomxTools (v3.2.0) and NanoStringNCTools (v1.6.1). To analyze the normalized counts of each protein, we used the GraphPad Prism 10 program (La Jolla, CA), specifically using unpaired t test and Mann-Whitney analysis. Comparisons for individual gene expression were measured as a log2 fold-change (FC). A linear mixed model was used to fit the normalized RNA count data to identify differentially expressed (DE) genes. The adjustment method to control false discovery rate (FDR) for multiple testing used in this study was the Benjamini and Hochberg (1995) method.^16^ The analysis was carried out using lme4 package (v1.1.31) and stats package (v4.1.2) with emmeans (v1.8.4.1).

## 3. RESULTS

### 3.1. Subject Demographics of CMV-Infected and Control Placentas

**Figure 1.**
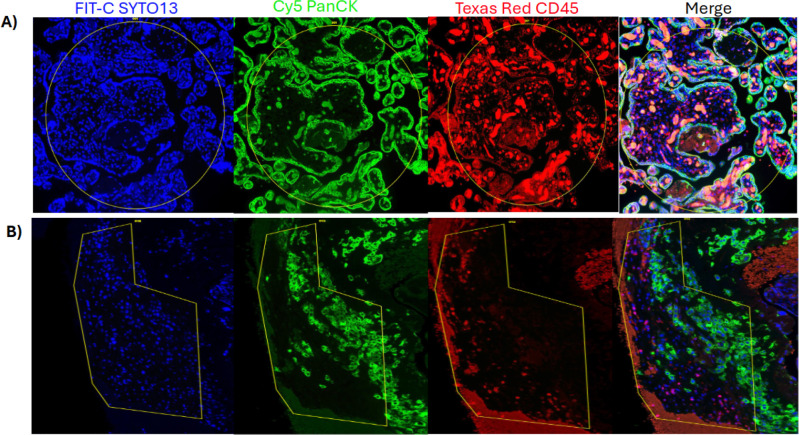
Representative Images of a CMV positive placental ROI from NanoString GeoMx Digital Spatial Profiler. CD45^+^ cells are shown in red, PanCK^+^ cells are shown in green, and nuclei (SYTO13) shown in blue. (A) Villous tissue. (B) Decidual tissue.

FFPE placental tissue was obtained from the Mayo Clinic Tissue Repository. Only participants who underwent Cesarean delivery were selected to minimize possible effects from the microbial environment during a vaginal delivery. For transcriptomic and proteomic analyses, we selected samples from women documented to have primary CMV infection (CMV^+^) during pregnancy (n = 3). Comparisons were made with CMV^−^ control samples of a similar gestational age (n = 4). The selected samples had comparable maternal ages, BMI, gestational age at delivery, and neonatal APGAR scores between groups (Table 1). Infants born to people with CMV infection had a lower median birth weight than those from control subjects (1.64 vs 2.99 kg, p= 0.05). Hematoxylin and eosin images and CMV immunohistochemical staining is shown in Supplemental Figures 1 and 2. Representative immunofluorescent images illustrate the villous and decidual ROIs collected in a CMV^+^ sample (Figure 1A and 1B).

### 3.2. Altered Gene Expression in CD45^+^ Immune Cells Exposed to CMV

**Figure 2.**
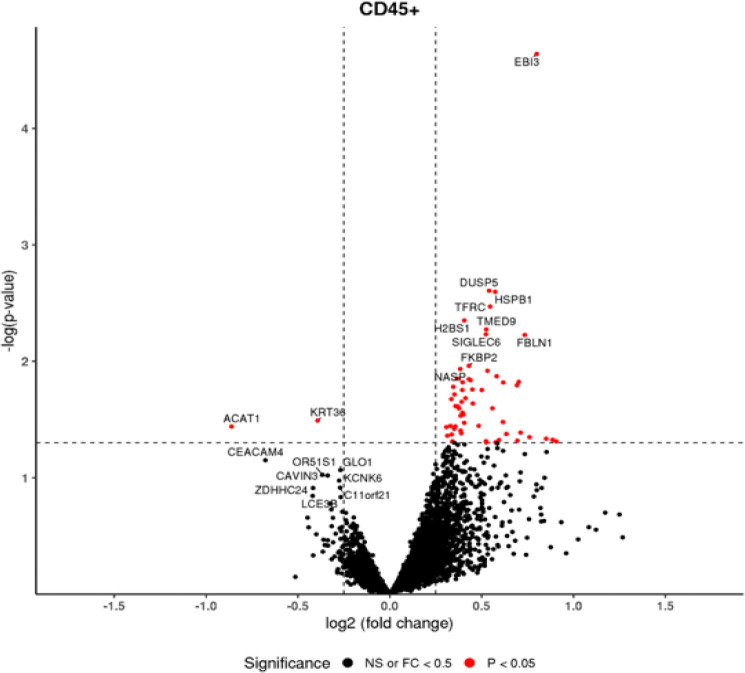
Differentially expressed genes from CD45^+^ immune cells exposed to CMV compared to controls. Red dots are genes that are significantly up- or down-regulated with a p value of 0.05 and fold change of 0.25 or higher. Black dots represent genes that are not significantly altered. Horizontal dotted line represents a −log(p-value) of 1.3. Vertical dotted lines represent significant thresholds for log2 fold changes of −0.25 and 0.25.

To gain insight on the molecular alterations that CMV infection may cause, we evaluated the transcriptomic profiles of immune cells within CMV^+^ placental tissue versus control placental tissue to identify which genes were differentially expressed. In CD45^+^ immune cells, we observed 63 differentially expressed genes (DEGs), of which 61 genes were significantly upregulated, and 2 genes were downregulated during CMV infection compared to no CMV infection (Figure 2). The DEGs in CD45^+^ immune cells were enriched in numerous immune system activation pathways using Ingenuity Pathway Analysis (IPA; Qiagen), including cachexia signaling, Class I MHC-mediated antigen processing and presentation, neutrophil extracellular trap signaling pathway, and neutrophil degranulation (Table 2). In addition to canonical pathways, our IPA analysis gave data on upstream regulators. One of the top upstream regulators for CMV-exposed immune cells was CD40L. Additionally, genes from CD45^+^ immune cells exposed to CMV infection upregulate pathways implicated in pro cell death, such as immunogenic cell death pathway, death receptor signaling, and granzyme A signaling. Pathways related to sex hormones and growth factors were also altered in CD45^+^ immune cell populations during CMV infection, such as estrogen receptor signaling, extra-nuclear estrogen signaling, and growth hormone signaling. Together, these data suggest that processes critical for healthy pregnancy are altered during CMV infection.

### 3.3. Gene Expression in Trophoblasts Exposed to CMV

**Figure 3.**
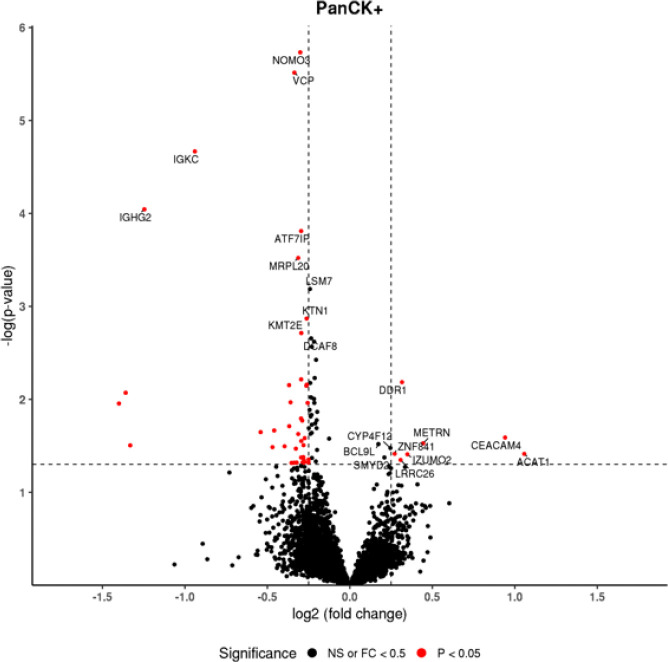
Differentially expressed genes in trophoblasts exposed to CMV. Red dots are genes that are significantly up- or down-regulated with a p value of 0.05 and fold change of 0.25 or higher. Black dots represent genes that are not significantly altered. Horizontal dotted line represents a −log(p-value) of 1.3. Vertical dotted lines represent significant thresholds for log2 fold changes of −0.25 and 0.25.

Next, overall changes in trophoblast gene expression were analyzed between CMV^+^ and CMV^−^ placentae. We identified 75 DEGs in trophoblasts from CMV infected placentae compared to non-infected controls, with 68 genes downregulated, and 7 genes upregulated (Figure 3). The corresponding IPA of these trophoblasts revealed that immune response related pathways were decreased in trophoblasts exposed to CMV, including those genes involved in the complement cascade, NFAT regulation, and communication between innate and adaptive immune cells (Table 3). Furthermore, pathways implicated in maternal-fetal tolerance were also decreased in CMV^+^ trophoblasts, specifically allograft rejection-, prolactin-, and TGF-b-signaling.

### 3.4. Upregulation of Immune Activation Proteins and Downregulation of Cell Death Proteins in CD45^+^ Immune Cells during CMV Infection

**Figure 4.**
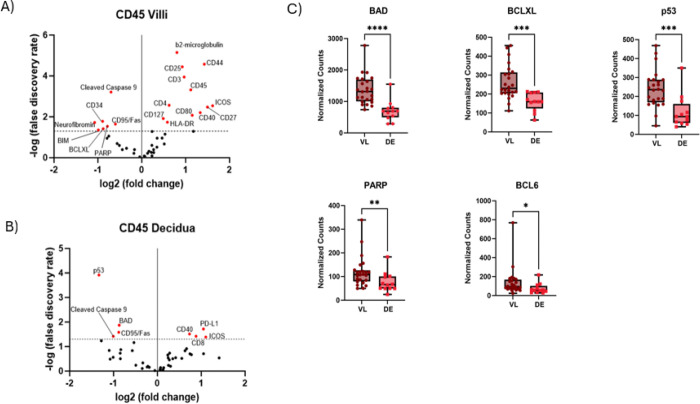
Comparison of protein abundance in CMV^+^ tissue versus control in immune cell population. A) Protein abundance in CD45^+^ cells in villous tissue. B) Protein abundance in immune cells in villous tissue. C) Protein counts graphed by tissue location in the CMV^+^ placenta. Mann-Whitney statistical test illustrated as box and whisker plot. VL = Villi, DE = Decidua

To better understand the cellular and location-specific effects of CMV on placental immunity, we analyzed the phenotypes of CD45^+^ cells in the placental villi and decidua. There was a significant increase in CD4^+^ T cells within the villous tissue having an activated phenotype during CMV infection compared to controls (Figure 4A). These activation markers included: ICOS, CD25, CD27, CD40, CD44, HLA-DR and CD80. Conversely, numerous proteins related to apoptosis were downregulated in immune cells in the villi during CMV infection compared to immune cells in the decidua. In decidual immune cells, there was an increase in CD8^+^ cytotoxic T cells expressing activation receptors CD40 and ICOS, and the checkpoint molecule PD-L1 during CMV infection compared to controls (Figure 4B). Additionally, we observed that cell death proteins were significantly downregulated in immune cells in the decidual compartment during CMV infection compared to immune cells in the villi. We then sought to identify differences in apoptotic proteins used by CD45^+^ cells by location in the placenta, specifically in villous tissue (VL) compared to the decidua (DE). We observed that 5 cell death markers had a significantly higher abundance on CD45^+^ cells in the VL compared to the decidua from tissue infected with CMV (Figure 4C). These included BAD (1364 vs. 699.1, p<0.0001), PARP (119.4 vs. 76.76, p=0.0088), BCL6 (p=0.0164), BCLXL (261.4 vs. 159.6, p=0.0003), and p53 (239.9 vs. 118.7, p=0.0006), respectively. Overall, these data indicate that cell death markers were decreased in immune cells from CMV infected placentae and spatially, the immune cells in the villi expressed more cell death markers than the decidua.

### 3.5. Protein Expression Changes in Trophoblasts Exposed to CMV

**Figure 5.**
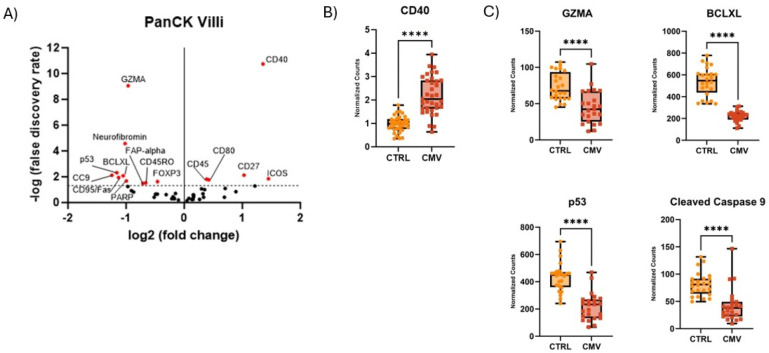
Comparison of proteins on trophoblasts from CMV^+^ placental tissue versus control placental tissue. A) Linear Mixed Model statistical tests illustrated as volcano plot. PanCK^+^ cells in villous tissue. B) Normalized counts of immune activation marker CD40 in PanCK^+^ cells illustrated in box and whisker plots. C) Normalized counts of apoptosis markers in PanCK^+^ cells illustrated in box and whisker plots.

Next, we analyzed protein expression in PanCK^+^ trophoblast cells from CMV^+^ placental tissue. The trophoblasts of CMV^+^ tissue displayed significant upregulation of activation markers, including CD40, CD27 and CD80 compared to uninfected placentae (Figure 5A and B). To confirm that these cells express CD40, we performed additional immunohistochemistry and showed that these cells express high levels of CD40 on their surface (Supplemental Figure 2). We also observed a significant downregulation of cell death markers on trophoblasts exposed to CMV infection compared to no infection (Figure 5C), including decreased GZMA (31.24 vs. 73.34, p<0.0001), BCLXL (210.7 vs. 538.2, p<0.0001), p53 (164.1 vs. 434.2, p<0.0001), and cleaved caspase 9 (31.05 vs. 81.45, p<0.0001), respectively. In all, trophoblasts during CMV infection upregulate activation markers while downregulating apoptotic proteins.

## 4. DISCUSSION

The overall aim of our study was to identify transcriptomic and proteomic alterations in immune and trophoblast cells in the placenta resulting from CMV infection. Our study adds the unique element of comparing the effects of CMV infection spatially on distinct regions within the placenta, namely the placental villi and decidua.

We found that CMV exposed immune cells downregulated numerous genes and proteins related to pathways critical for immune activation and cell death compared to controls. Previous studies have shown that CMV encodes a gene called UL36, which suppresses the expression of CD95/Fas by inhibiting caspase 8 activation.^17^ Our data also showed a decrease in CD95/Fas in immune cells infiltrating in CMV^+^ placentae. Additionally, CMV had an intricate effect on the unfolded protein response, as it activated and inhibited genes in the pathway to favor viral replication.^18^ The multifaceted effect of CMV infection on the unfolded protein response suggests that the overall enrichment of the pathway might be bidirectional, thus resulting in a z-score of 0. Moreover, pathways that are essential in the process of a successful pregnancy were also dysregulated with a z-score of 0, namely growth hormone signaling, prolactin receptor signaling, estrogen receptor signaling and extra-nuclear estrogen signaling. Estrogen and its receptors are critical during pregnancy to facilitate placentation, uterine growth, and fetal organ development.^19–21^ Therefore, dysregulation in either direction can contribute to adverse pregnancy outcomes in the context of CMV infection.

Trophoblasts are critical for maintaining a healthy and functional placenta.^22^ Trophoblasts exposed to CMV however, displayed a distinct mRNA and protein profile, including downregulation of pathways implicated in cell death, including phagosome formation and Fc gamma receptor-dependent phagocytosis. Since phagocytosis is critical for clearance and eventual cell death of infected cells,^23,24^ CMV may downregulate the process of phagocytosis to evade the host immune response and persist in infected cells. Similar to our immune cell transcriptomic results, several pathways that dictate pregnancy outcomes are dysregulated in CMV-exposed trophoblasts, including the activin and inhibin pathway, S100 family signaling pathway, and Transforming Growth Factor-beta (TGF-beta) signaling. The activin and inhibin pathway is involved in the differentiation, migration, and invasion functionality of trophoblasts.^25^ Intrauterine growth restriction and pre-eclampsia are just two of the many adverse pregnancy outcomes that can result from an aberration of these hallmark trophoblast functions.^26^ Moreover, these two adverse outcomes have been shown to be consequences of CMV infection.^27,28^ S100 proteins are important during embryonic development, as they are involved with implantation, intrauterine growth, and fetal brain development.^29^ This is consistent with the observed fetal consequences of cCMV, which includes intrauterine growth restriction and preterm birth.^30^ Lastly, TGF-beta plays an integral role in pregnancy; not only is it implicated in placental development, hormonal secretion, and embryonic growth and development, but it also regulates maternal-fetal interactions by creating a more immune-tolerant environment for the allogenic fetus to develop in.^31^ Disruption of TGF-beta signaling suggests a mechanism for miscarriage and preterm birth that results from CMV infection during pregnancy. Overall, the dysregulated pathways and their corresponding genes in CMV exposed trophoblasts showed a loss of immune suppression mechanisms and dysregulation of trophoblast function, which may consequentially allow CMV to more easily replicate and evade the host immune response.

Guided by our transcriptomic data, we observed a distinct proteomic profile in the CMV exposed immune cell population that parallels our mRNA data. First, we saw a significant increase of immune activation proteins. Specifically, CD4^+^ T cells were more abundant in immune cells of the villi of CMV^+^ placentae compared to the immune cells of the decidua. Additionally, the corresponding co-stimulatory molecules ICOS, CD40, CD44, CD80 and HLA-DR, an MHC class II surface receptor, were significantly increased in CMV^+^ placental immune cells. This CD4^+^ predominance contrasts with our previous analysis of placentae diagnosed with villitis of unknown etiology, which exhibited a CD8 bias.^32^ In the decidua, there were more CD8^+^ T cells compared to CD4^+^ T^+^ cells, however, PD-L1 was also higher in the decidua. This suggests that T cells in the decidua are likely to maintain immune tolerance unlike those in the villi, consistent with previous literature.^33^ Additionally, we observed that Beta-2-microglobulin (B2M), a component of the MHC class I molecule, was highly abundant in the villous compartment of the placenta. Other investigators have shown that nucleated cells expressing B2M are either virally-infected or engaged in antigen presentation to CD8^+^ T cells.^34^ Studies have also shown that CMV can displace B2M from HLA Class I MHC and use the remaining molecule as a viral receptor.^34^ Alterations in immune activation proteins are consistent with the alteration of immune signaling pathways that we noted in our transcriptomic study. Conversely, we only saw 4 proteins significantly altered in CMV exposed immune cells of the decidua, which suggests that the majority of the CMV response occurs in the villi by infiltrating immune cells. Moreover, PD-L1, a checkpoint inhibitor of T cells, is highly abundant on CD45^+^ immune cells in the decidua, compared to ICOS, a costimulatory molecule expressed on T cells, which was upregulated in CD45^+^ cells infiltrating into the villi. Our results shed new light on mechanisms triggered by CMV in the placenta and indicate that different immune responses are occurring in the villi compared to decidua.

CMV has been shown to manipulate apoptotic processes to survive and replicate in the cells that it infects.^35^ Previous CMV infection findings that show that the virus encodes proteins that mediate an anti-apoptotic effect on infected cells.^36^ Our data reflected these observations, as CMV-exposed immune cells had significantly less cell death related proteins and showed pivotal alterations in apoptotic pathways. We noted both intrinsic and extrinsic apoptotic markers were decreased, including BIM, BCLXL, Cleaved Caspase 9 and CD95/Fas. In addition, markers related to DNA damage and repair were also less abundant in CMV exposed immune cells in the villi, including PARP and neurofibromin. Neurofibromin has been shown to render cells more susceptible to apoptosis through Ras pathway signaling.^37^ Overall, we noted that more cell death proteins were significantly decreased in CD45^+^ cells infiltrating into placental villi than into the decidua. Again, this may suggest that the virus preferentially impacts villous infiltrated immune cells to replicate in as opposed to immune cells in the decidua, as previous studies have concluded.^38^ Other cell death markers were more abundant in villous infiltrated immune cells than in decidual immune cells, including BAD, BCLXL, and p53. BCLXL is an anti-apoptotic protein, which regulates the viability of a virally-infected cells.^39^ Studies using the Epstein-Barr virus (EBV), another member of the Herpesviridae, have shown that EBV-related proteins upregulate expression of BCLXL.^40^ Our data suggests that due to the under abundance of apoptotic markers in villous immune cells of CMV^+^ placentae, CMV may preferentially impact survival of cells in villous tissue more than those in the decidua.

We identified a distinct proteomic profile in trophoblast cells from CMV^+^ placentae. Similar to our immune cell population, the villous trophoblasts were more activated and less apoptotic compared to control trophoblasts. Specifically, CD40 was the most highly abundant protein in CMV exposed trophoblasts. The increase of CD40 suggests an increase in immune activation, which is a consistent finding in studies investigating the effects of CMV infection on endothelial cells.^41^ These findings generate further questions about immune cell and trophoblast interactions during CMV infection, such as whether their interaction is overall helpful towards the protection of the fetus from the virus, or detrimental due to the associated inflammation the interaction may induce. In addition, we noted a similar pattern of fewer cell death markers in trophoblasts, less neurofibromin, BCLXL, PARP, p53, cleaved caspase 9, and CD95/Fas. Unique to trophoblasts, CMV decreases GZMA, FOXP3, and FAP-alpha. Granzyme A (GZMA) is a serine protease that is released by cytotoxic T cells and natural killer (NK) cells, and studies have shown that CMV produces a protein, UL36, that decreases GZMA expression,^42^ protecting CMV infected cells and evading host defense mechanisms.^43^ FOXP3 has been shown to be selectively expressed by trophoblasts, and its expression differs at different time points during pregnancy.^44^ FOXP3 is important for the invasion and proliferation of trophoblasts during the first trimester, and reduced protein expression was noted in placental tissue from recurrent pregnancy losses.^44^ The decrease in abundance of FOXP3 in trophoblasts has been hypothesized as a physiologic change in third trimester placenta, promoting the pro-inflammatory process of labour. Finally, fibroblast activated protein alpha (FAP-alpha) is an important protein in early pregnancy, as it is involved in decidualization and tissue remolding during implantation.^45^ CMV has been shown to downregulate matrix metalloproteinases, which are important enzymes that trophoblasts use to invade the uterine wall.^46^ Thus, early CMV infection may decrease FAP-alpha resulting in intrauterine growth restriction or spontaneous abortion, as these outcomes are well established consequences of fetal infection.

While our study revealed new potential mechanisms that CMV could utilize to infect and replicate in the placenta, there were limitations encountered. First, our sample sizes were small. Currently CMV screening is not standard of care for immunocompetent hosts, including those who are pregnant. Therefore, only CMV infections that cause severe fetal and/or maternal symptoms have traditionally been captured. Recently, the state of Minnesota passed legislation to begin universal CMV screening in neonates, which will widen the sample pool for further investigations of cCMV and provide better data to define the true incidence of this disease during pregnancy. Additionally, we could not access the neonatal medical records for this study. Our analysis was therefore restricted to maternal medical records, making it harder to make definitive statements on the presence and severity of cCMV signs and symptoms in neonates following delivery. Future directions include increasing sample size and examining these findings with functional assays to confirm downregulation of cell death mechanisms, which will more widely characterize trophoblast-immune cell interactions during CMV infection.

## 5. CONCLUSION

The placenta has been implicated as a pathway of vertical transmission of CMV, but more work is necessary to uncover the exact mechanisms the virus uses to circumvent the protection that the placenta should provide to the developing fetus. Due to the devastating long-term clinical effects of cCMV, it is paramount that we continue to characterize the pathobiology of the virus that contribute toward the identification of biomarkers, creation of new and effective treatments, and the development of an effective vaccines against CMV. Overall, our data highlights new proteins and pathways which are altered in CMV^+^ placental tissue that may be important towards understanding the pathogenesis of CMV infection during pregnancy.

## Figures and Tables

**Table 1.** Patient demographics among the CMV+ and control placentae utilized in this study.

	CMV+ (n=3)	Control (n=4)	Total (n=7)	P-value
Maternal Age (years)	28 (24–32)	28.8 (23–38)	28.4 (23–38)	0.7714
Pre-gravid BMI (kg/m^2^)	34.3 (28.7–41.0)	27.4 (19.0–29.4)	30.3 (19.0–41.0)	0.4000
Gestational age (weeks + days)	33+2 (31+1 – 34+6)	37+6 (33+5 – 39+2)	35+6 (31+1 – 39+6)	0.1714
Birth weight (kg)	1.64 (1.04–2.05)	2.99 (2.4–3.30)	2.41 (1.04–3.30)	0.0571
APGAR Score at 5 mins	8 (6–9)	8 (6–9)	8 (6–9)	>0.9999

**Table 2.** Pathways altered in CD45^+^ immune cells exposed to CMV. Alterations in the gene expression of CMV exposed immune cells versus control immune cells correspond to changes in gene pathways using Ingenuity Pathway Analysis.

	−log(p-value)	Ratio	z-score	Molecules
Cachexia Signaling Pathway	1.79	0.0112	2	CAPN6,HSPA5,INHBA,PRKCZ
Class I MHC mediated antigen processing and presentation	1.72	0.0107	2	CTSL,HSPA5,PDIA3,VAMP8
Neutrophil Extracellular Trap Signaling Pathway	1.69	0.0104	2	NDUFA10,NDUFA13,PDIA3,PRKCZ
Neutrophil degranulation	1.39	0.0084	2	MIF,PAFAH1B2,VAMP8,VCP

**Table 3.** Pathways Altered in trophoblasts exposed to CMV. Alterations in the gene expression of CMV exposed trophoblasts versus control trophoblasts correspond to changes in gene pathways using Ingenuity Pathway Analysis.

	−log(p-value)	Ratio	z-score	Molecules
Activin Inhibin Signaling Pathway	6.58	0.0345	−2.646	EP300,IGHG1,IGHG2,IGHG3,IGHG4,IGKC,SMAD2
IL-12 Signaling and Production in Macrophages	6.31	0.0315	1.134	EBI3,EP300,IGHG1,IGHG2,IGHG3,IGHG4,IGKC
Fcgamma receptor (FCGR) dependent phagocytosis	5.95	0.0382	−1.633	AHCYL1,IGHG1,IGHG2,IGHG3,IGHG4,IGKC
Role of Macrophages, Fibroblasts and Endothelial Cells in Rheumatoid Arthritis	5.28	0.022	−2.449	IGHG1,IGHG2,IGHG3,IGHG4,IGKC,MIF,RAP1A
Neutrophil Extracellular Trap Signaling Pathway	4.75	0.0183	−2.646	IFNAR1,IGHG1,IGHG2,IGHG3,IGHG4,IGKC,NDUFA13
Macrophage Alternative Activation Signaling Pathway	4.36	0.0278	−2.236	IGHG1,IGHG2,IGHG3,IGHG4,IGKC
Dendritic Cell Maturation	3.76	0.0156	−2.236	IFNAR1,IGHG1,IGHG2,IGHG3,IGHG4,IGKC
Phospholipase C Signaling	3.33	0.0108	−2.449	EP300,IGHG1,IGHG2,IGHG3,IGHG4,IGKC,RAP1A
Phagosome Formation	2.5	0.00894	−2.236	IGHG1,IGHG2,IGHG3,IGHG4,IGKC,RAP1A
Communication between Innate and Adaptive Immune Cells	2.49	0.0108	−2.236	IGHG1,IGHG2,IGHG3,IGHG4,IGKC
S100 Family Signaling Pathway	2.27	0.00802	−2.449	IGHG1,IGHG2,JGHG3,IGHG4,IGKC,SMAD2

## Data Availability

Spatial transcriptomics data presented in this manuscript can be access on the NCBI GEO platform under GSE282298
